# Electrogram voltage and pacing threshold before ablation, measured by mini-electrodes, predict parameters indicative of transmural lesions in the human atrium

**DOI:** 10.1007/s10840-019-00539-6

**Published:** 2019-05-02

**Authors:** Carla Lázaro, Teresa Barrio-López, Eduardo Castellanos, Mercedes Ortiz, Martín Arceluz, Jesús Almendral

**Affiliations:** Electrophysiology Laboratory and Arrhythmia Unit, Hospital Madrid Monteprincipe, Grupo HM Hospitales, University CEU-San Pablo, Avda. Montepríncipe, 25 28660 Madrid, Boadilla del Monte Spain

**Keywords:** Ablation, Radiofrequency lesion, Mini-electrodes

## Abstract

**Purpose:**

An important attenuation of the atrial signal recorded with mini-electrodes (ME) embedded in an 8-mm tip was associated with a transmural radiofrequency lesion. Our aim was to assess if parameters obtained from ME or conventional bipoles before applications predict successful atrial lesions.

**Methods:**

We prospectively included 33 consecutive patients undergoing cavotricuspid isthmus (CTI) ablation. Electrogram voltages and pacing thresholds were measured with ME and conventional bipoles before and after radiofrequency (RF) applications. The time before the loss of capture during applications was recorded. Lesions were considered successful, in accordance with preclinical data, if ME voltage decreased > 54%.

**Results:**

Of 207 applications, 107 could be analyzed. During applications, voltages decreased more in the ME than in the conventional bipoles (66.8 ± 26.1% vs 37.5 ± 42.5%, *P* = 0.001). Likewise, pacing threshold increased significantly more using the ME (86.3 ± 22.9% ME, 52.6 ± 35.6% conventional, *P* = 0.001). ME pre-ablation voltages were significantly higher and pacing thresholds significantly lower in successful lesions (voltage 0.88 ± 0.71 vs 0.26 ± 0.18 mV, *P* = 0.0001; threshold 1.6 ± 1.7 vs 2.8 ± 3.0, *P* = 0.04). Neither of these parameters with conventional bipoles nor time to loss of capture showed differences. A ME voltage > 0.33 mV and a pacing threshold < 1.5 mA predicted a successful lesion with 0.78 and 0.6 sensitivity and 0.78 and 0.59 specificity.

**Conclusions:**

Certain pre-ablation parameters derived from ME such as electrogram voltage and pacing threshold differ from those obtained by a conventional configuration and can predict a successful atrial lesion.

One of the limitations of using a radiofrequency (RF) catheter ablation as therapy for cardiac arrhythmias is that the size of the lesions produced in the heart is unknown. Using regular-sized electrodes, two parameters have been studied in animal models as indicators of lesion size: a decrease in electrogram voltage and an increase in pacing threshold [1, 2]. The former was modestly related to lesion size in atrial tissue [1] and had no significant correlation to lesion size in the ventricle [2]. The latter showed a more accurate correlation with lesion size in ventricular tissue [2].

The advent of catheters with mini-electrodes (ME) embedded in a larger ablation tip has provided the opportunity to test whether the information obtained from these ME correlates with the lesion produced by a regular ablation tip. In a canine model in which 95% of atrial lesions were transmural after RF lesions of 60 s, the electrogram voltage of the ME bipolar electrogram decreased by a mean of 82 ± 14% [3].

However, the above parameters can only be obtained after the lesion has been created, and in the clinical setting, it would be desirable to predict an appropriate lesion size before or at the beginning of the RF application. Thus, we aimed to study a number of parameters that can be obtained both with standard electrodes and ME before or at the beginning of the RF application.

In the present study, we analyzed the extent to which electrogram voltage and pacing threshold prior to a RF application and time to loss of capture during ablation are able to predict findings of a successful lesion as previously described for the ME in atrial tissue [3].

## Methods

### Study design

This is a prospective observational study in which RF applications were delivered in the conventional way according to the best clinical practice. However, a number of parameters were measured before, during, and after each application (see below). We took advantage of the previous preclinical observation of a reduction in the voltage of the ME bipolar electrogram by a mean of 82 ± 14% in transmural lesions [3] and considered that reductions of ≥ 54% [82%—2 standard deviation (SD)] were likely to predict successful lesions (“probably transmural”). This allowed for a posterior categorization of RF lesions as likely successful or likely unsuccessful. In addition, the effects of each RF lesion on the parameters of the conventional bipolar configuration (tip to ring) were compared to those on the ME.

The study was approved by the Ethics Committee of our Institution and patients provided a written copy of informed consent for their participation in the study.

### Patient population

Consecutive patients referred to our Institution for RF ablation of the cavotricuspid isthmus (CTI) were included if they met the following inclusion criteria: (1) at least one spontaneous episode of documented typical atrial flutter, defined by an inverted sawtooth pattern in the inferior ECG leads and a regular atrial rate > 240 bpm or sustained isthmus-dependent atrial flutter induced during an electrophysiologic study in a patient with known or suspected atrial arrhythmias; (2) ablation was performed with an 8-mm tip ablation catheter having ME embedded at its tip (Intella tip MIFI, Boston Scietific); (3) the study was performed under general anesthesia.

Exclusion criteria were the following: (1) previous CTI ablation procedure, (2) initiation of sustained atrial fibrillation during the ablation procedure, (3) technical difficulties in obtaining catheter stable positions that could allow the measurements of the required parameters, (4) concomitant pulmonary vein isolation procedure, and (5) pregnancy.

### Electrophysiological study and RF ablation

Studies were performed in the postabsortive state and under general anesthesia. A “deflectable halo” catheter (2–10–2 mm interelectrode distance; Livewire 7F St Jude) was placed along the tricuspid annulus (TA). A quadripolar catheter (5F Torqr, Medtronic, or 7F MarinR, Medtronic) was placed at the coronary sinus. Ablation was performed with an ablation catheter with ME embedded in a 8-mm tip (Intella Tip MIFI).

If the patient was in spontaneous or induced atrial flutter, and mapping and entrainment techniques characterized the arrhythmia as common atrial flutter, ablation was initiated during flutter. Otherwise, the ablation procedure was performed during sinus rhythm. RF applications were delivered point by point in the temperature-controlled mode with a power limit of 70 W, starting with a temperature limit of 55 °C and increasing the temperature up to 65 °C if the power did not reach at least 40 W. The lesion set included successive RF applications along a theoretical line crossing the CTI midway between its septal and lateral aspect as seen in the 45° left anterior oblique fluoroscopic projection, starting at its ventricular side and terminating at its caval end. After the delivery of each application, the catheter was carefully moved under fluoroscopic guidance to another position as stable as possible, approximately 6–8 mm closer to the caval end of the CTI as judged by the 30° right anterior oblique fluoroscopic projection. Each fluoroscopic catheter position in which an RF application was delivered was “frozen” in a second fluoroscopic screen for reference.

After the completion of the RF ablation line or when atrial flutter terminated, conduction across the CTI was evaluated by analyzing the activation sequence of the multipolar catheter along the tricuspid annulus during coronary sinus pacing. If conduction persisted, conduction gaps were searched for by exploring electrical signals along the ablation line during sinus rhythm or coronary sinus pacing, and additional RF lesions were delivered. If at any time during this part of the procedure, the operator considered that conduction persisted because the delivered power was insufficient, the catheter was substituted by an irrigated tip catheter. The procedure finished when bidirectional conduction block across the CTI was achieved and persisted for at least 30 min.

### Clinical follow-up

Patients were followed up for at least 6 months in our outpatient clinic, or they were in-touch via telephone (in one case). The symptomatic and clinical status was evaluated along with a 24-h Holter monitoring and a 12-lead ECG.

### Study measurements

The following data were obtained: (1) bipolar pacing thresholds with both the conventional configuration (tip to ring) and the ME before and after each RF application; (2) bipolar electrogram amplitudes of both the conventional configuration (tip to ring) and the ME before and after each RF application; (3) times to loss of capture during RF energy delivery. In order to obtain these measurements, pacing from the ME with a voltage output 10 times the diastolic threshold was started prior to RF delivery and maintained until loss of capture. All measurements and stimulation from the ME were performed from the bipole with the largest amplitude. Additionally, the mean power of each application was recorded.

The “gold standard” for the creation of a successful (probably transmural) lesion, based on preclinical data [3], was a decrease in the electrogram amplitude of the ME > 54% (mean–2 SD) of % decrease in electrogram amplitude of the preclinical study).

### Statistical analysis

For the main analysis, only RF applications of the first ablation line were considered, and only those in which the catheter remained in a stable position as judged by fluoroscopy were evaluated. The values are expressed as median ± SD. The Spearman and the Pearson correlation coefficients were calculated for continuous variables. The correlation was considered strong when the correlation coefficient (Pearson *R* or Spearman Rho) was more than 0.70, moderate when the correlation coefficient was more than 0.30 and less than 0.70, and weak when the correlation coefficient was less than 0.30. We selected a cut-off point of reduction in voltage ≥ 54% for successful ablation lesion [3]. Receiver operator curves (ROC) were constructed to determine the area under the curve (AUC) and the best cut-off values for each pre-ablation predictive parameter (electrogram voltage and pacing threshold). These cut-off values were used to calculate the sensitivity, the specificity, the accuracy, and the negative and positive predictive values (NPV and PPV). Predictive parameters were also combined in an attempt to increase predictive accuracy. Sensitivity and specificity, the expected distribution of true and false positives, were estimated through sampling fractions. Continuous variables were studied using the Student’s *t* test. The *p* values were based on the two-tailed test, and *p* values < 0.05 were considered statistically significant.

## Results

The study group consisted of 33 patients whose clinical characteristics are summarized in Table 1.

**Table 1:** Clinical characteristics

No. cases	33
Age, years, mean ± SD	65.7 ± 8.5
Male, *n* (%)	25 (76)
Structural heart disease, *n* (%)	27 (9)
Hypertension, *n* (%)	15 (45)
Diabetes mellitus, *n* (%)	4 (12)
Left ventricular ejection fraction > 55%, *n* (%)	25 (75)
Atrial flutter, *n* (%)	13 (39)
Sinus rhythm, *n* (%)	20 (60)

In total, 207 RF applications were delivered. Of them, 138 were delivered as part of the first ablation line (a mean of 4.2 applications per patient) and were initially considered for analysis. The remaining 69 applications were delivered over gaps after the first ablation line was completed. Electrogram voltages and pacing thresholds with both the conventional configuration and the ME were measured before and after an RF application without catheter displacement at a previously non-ablated site in 107 RF applications, which constituted the basis of our analysis. The reasons for not analyzing the remaining applications were as follows: catheter displacement in 14 applications (6.8%) and catheter instability in 17 (8.3%). The mean delivered power was 24.1 ± 13.9 W. In seven cases, the operator decided to change to an irrigated tip catheter at some point during the procedure, always after the first ablation line had been performed.

Persistent (> 30 min) bidirectional conduction block of CTI was recorded in all cases.

Patients were followed up for at least 6 months in our outpatient clinic; in one case, the follow-up was made by telephonic contact. We evaluated the symptomatic and clinical status along with a 24-h Holter monitoring and a 12-lead ECG. There were no recurrences of common atrial flutter. Five (15.5%) patients recurred with an atrial tachyarrhythmia, 4 of them (12.1%) presented atrial fibrillation and 1 (3%) atypical atrial flutter.

### Changes in electrogram voltage and pacing threshold in relation to RF applications

**Fig. 1:**
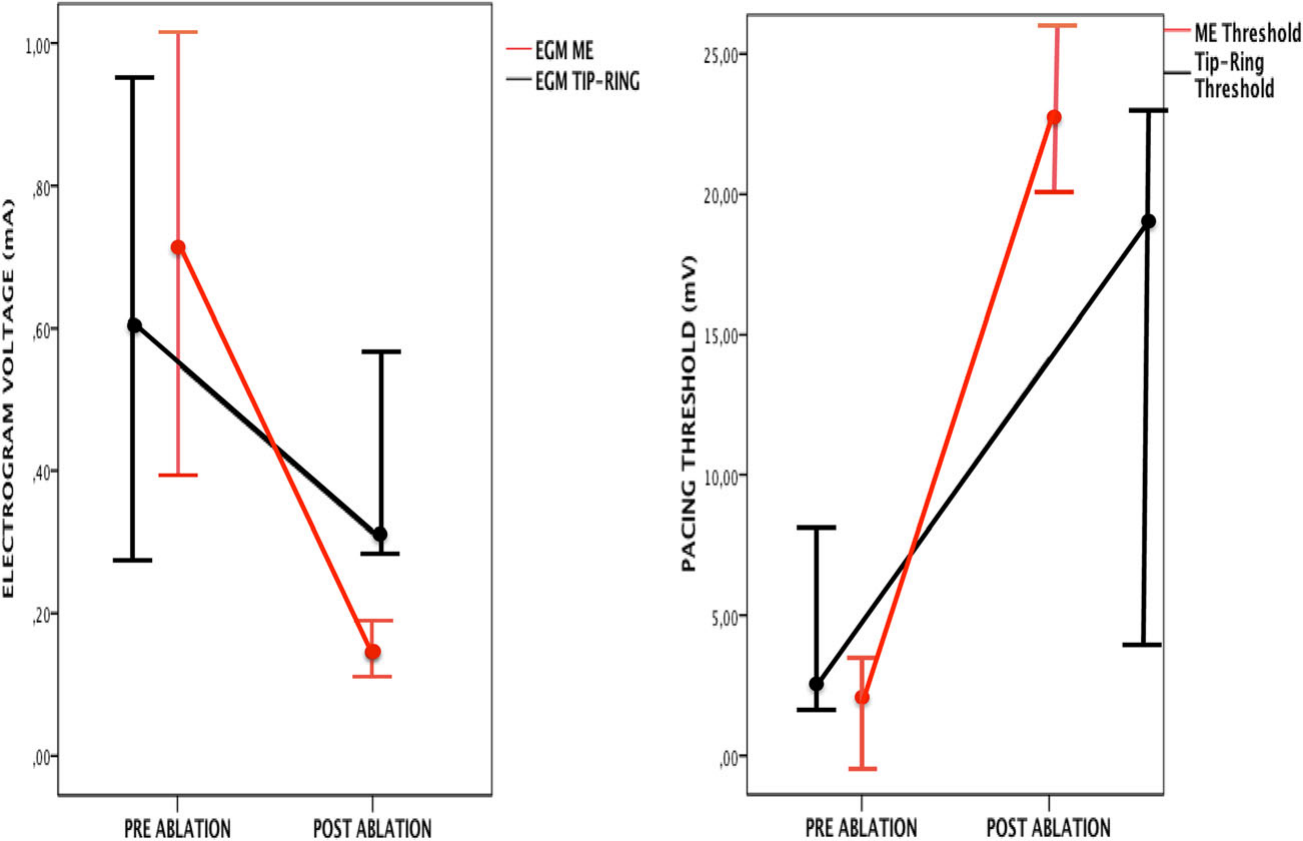
Electrogram (EGM) voltage and pacing thresholds pre- and post-ablation, as recorded from the mini-electrodes (ME) and from the conventional tip to ring configuration. Values represent mean and standard error of the mean. See text for discussion

**Fig. 2:**
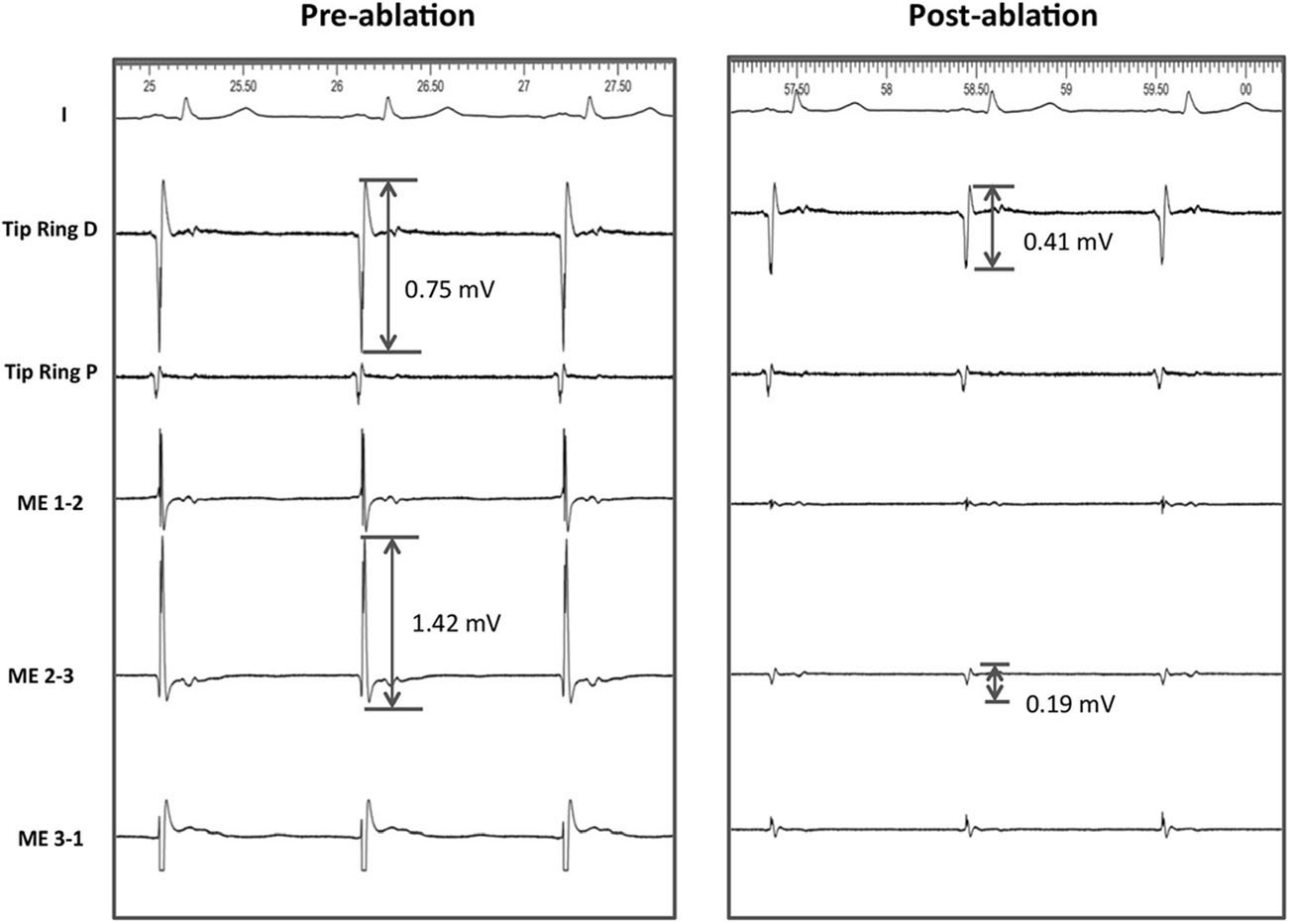
Example of reductions in electrogram voltage after RF applications. The tracings show lead II and bipolar recordings from the conventional electrodes (distal, D, proximal P) and the mini-electrodes (ME) pre- and post-ablation in the same patient and site. Note that pre ablation the voltage of the mini electrodes is higher than that of the conventional recordings. Also note that reductions are seen in all recordings but are more intense in the ME recordings (87% reduction in ME, 45% in the conventional distal bipole)

Pre-ablation, the ME exhibited greater electrogram voltage and lower pacing thresholds than the tip to ring conventional configuration (Table 2). In contrast, after RF ablation, the ME electrogram voltage was lower and the pacing threshold higher than the tip to ring conventional bipole (Table 2, Fig. 1). Figure 2 illustrates an example model representative of the type of findings often recorded.

**Table 2:** Changes in electrogram voltage and pacing threshold in relation to RF applications. Values are mean ± SD

	Conventional bipole	Mini-electrodes	*P* <
Pre-ablation electrogram voltage (mV)	0.55 ± 0.33	0.72 ± 0.68	0.023
Post-ablation electrogram voltage (mV)	0.31 ± 0.28	0.14 ± 0.1	0.001
Pre-ablation pacing threshold (mA)	4.7 ± 3.0	1.9 ± 2.1	0.001
Post-ablation pacing threshold (mA)	14.1 ± 9.7	22.5 ± 10.3	0.001
Percent reduction (pre-post ablation) in electrogram voltage	37.5 ± 42.5	66.8 ± 26.1	0.001
Percent increase (pre-post ablation) in pacing threshold	52.6 ± 35.6	86.3 ± 22.9	0.001

**Fig. 3:**
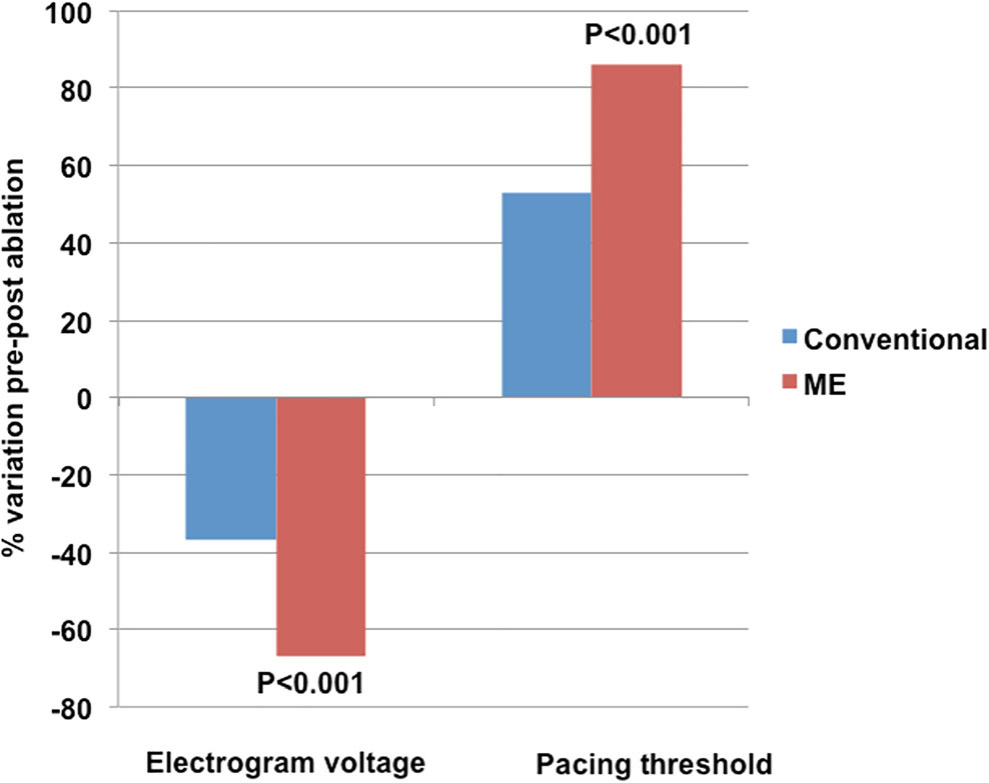
Reduction in electrogram voltage and increase in pacing threshold as a result of RF applications. Note that variations are significantly more important with mini-electrodes (ME) than with conventional (tip to ring) electrodes

The electrogram voltage of the ME was reduced by 0.58 ± 0.66 mV (66.8 ± 26.1%) and the electrogram voltage of the tip to ring bipole was also reduced (by 0.19 ± 0.56 mV, 37.5 ± 42.5%). However, the percentage of reduction was significantly higher for the ME (*P* < 0.001, Table 2, Figs. 2 and 3).

In an analogous fashion, the pacing threshold of the ME increased by 20.6 ± 10.4 mA (86.3 ± 22.9%) and that of the tip to ring bipole also increased (by 9.4 ± 9.0 mA, 52.6 ± 35.6%). However, the percentage of increase was significantly higher for the ME (*P* < 0.001, Table 2, Fig. 3).

Baseline electrogram voltage, as measured with ME, correlated significantly with the percent decrease in electrogram voltage that occurred with the RF applications (Spearman Rho = 0.7, *P* < 0.001, Pearson *r* = 0.5, *P* < 0.001). The correlation between the baseline pacing threshold and the percentage of decrease in electrogram voltage was negative and weak (Spearman Rho = − 0.23, *P* = 0.015, Pearson *r* = − 0.23, *P* = 0.015).

On the other hand, the correlation between the baseline electrogram voltage of the tip to ring bipole and the percentage of decrease in electrogram voltage was very weak (Spearman Rho: − 0.22, *P* = 0.8, Pearson *r* = − 0.10, *P* = 0.9). The correlation between the baseline pacing thresholds and the percent decrease in electrogram voltage was negative and weak (Spearman Rho, *r* = − 0.13, *P* = 0.15, Pearson, *r* = − 0.25, *P* = 0.009).

### Prediction of a successful lesion

**Fig. 4:**
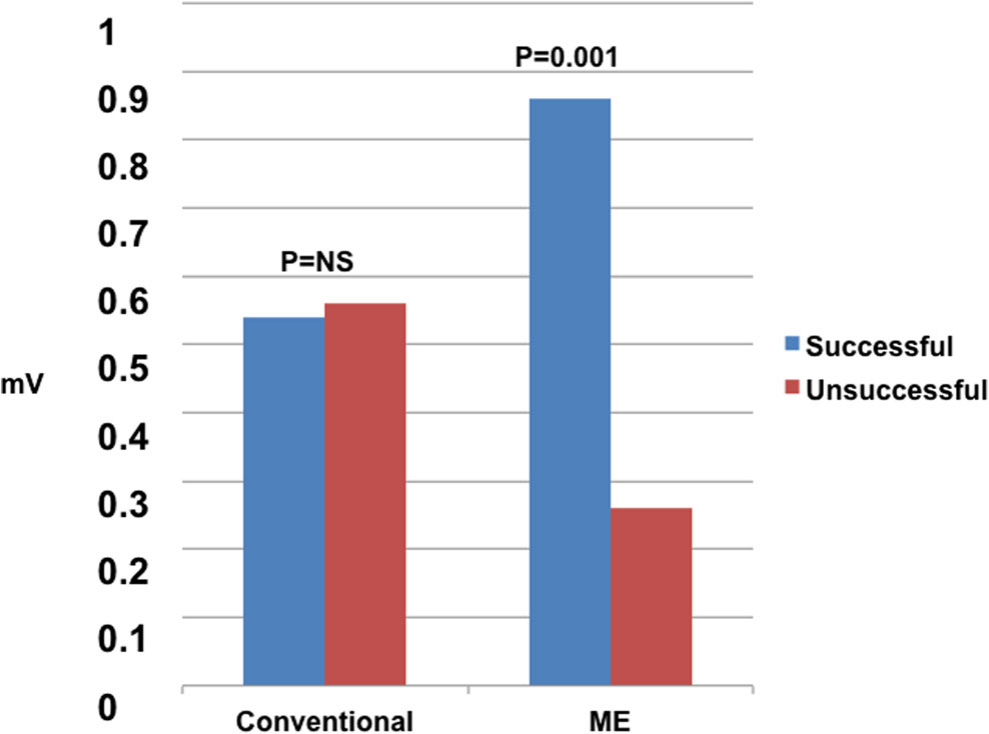
Pre-ablation electrogram voltage at lesions considered successful or unsuccessful. Note that there is lack of significant difference with the conventional configuration (tip to ring) vs a significant difference with mini-electrodes (ME)

**Fig. 5:**
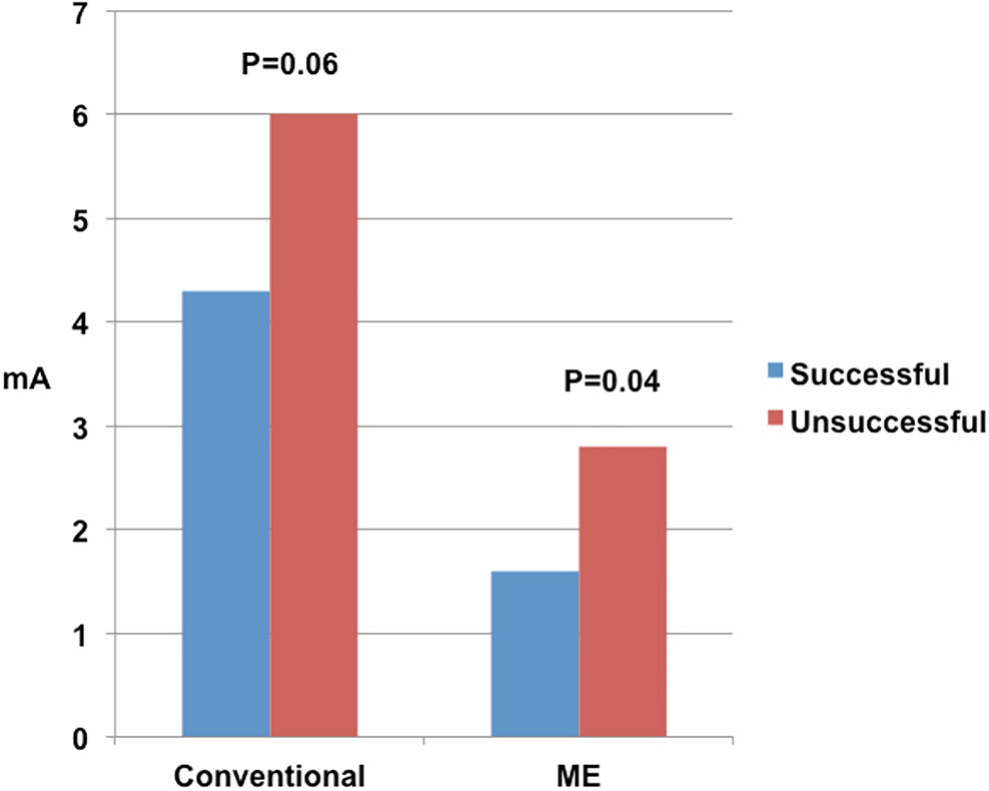
Pre-ablation pacing threshold at lesions considered successful or unsuccessful. Thresholds with the mini-electrodes (ME) were significantly lower at successful sites. Note that there is not a significant difference between the conventional configuration (tip to ring) and mini-electrodes (ME)

Overall, 80 of 107 RF applications (75%) were considered successful based on a reduction in the voltage of the ME bipole of ≥ 54%. In these lesions, the pre-ablation voltage of the ME bipole was significantly higher than in the remaining lesions (0.88 ± 0.71 vs. 0.26 ± 0.18, *P* < 0.001, Table 3, Fig. 4). Moreover, the pre-ablation pacing threshold of the ME bipole in successful lesions was significantly lower than in the remaining lesions (1.6 ± 1.7 vs 2.8 ± 3.0, *P* = 0.04, Table 3, Fig. 5).

**Table 3:** Pre-ablation electrogram voltages and stimulation thresholds in presumably successful and unsuccessful lesions

Lesion	Successful^a^	Unsuccessful	*P* =
No. (%)	80 (75)	27 (25)	N/A
Electrogram voltage, mini electrodes, mV	0.88 ± 0.71	0.26 ± 0.18	0.0001
Electrogram voltage, tip to ring, mV	0.54 ± 0.32	0.56 ± 0.33	0.9
Stimulation threshold, mini electrodes, mA	1.6 ± 1.7	2.8 ± 3.0	0.04
Stimulation threshold, tip to ring, mA	4.2 ± 2.2	6.0 ± 4.5	0.06
Time to loss of capture, s	9.9 ± 16.1	9.7 ± 8.6	0.9
Mean power, W	23.9 ± 13.2	24.7 ± 15.9	0.8

Values are mean ± SD

^a^Lesions resulting in a decrease in mini-electrode electrogram voltage ≥ 54% were considered successful (see text for discussion)

In contrast, the pre-ablation voltage of the conventional bipole (tip to ring) in successful lesions was not significantly different from the voltage in the remaining lesions (0.55 ± 0.33 vs 0.56 ± 0.32 mV, *P* = 0.9, Table 3, Fig. 4). Finally, the pre-ablation pacing threshold of the conventional bipole in successful lesions was not significantly different from the threshold in the remaining lesions (4.2 ± 2.2 vs 6.0 ± 4.54, *P* < 0.06) (Table 4, Fig. 5).

**Table 4:** Sensitivity, specificity, positive and negative predictive values (PPV, NPV), and accuracy for the best ME pre ablation voltage and the best ME pre-ablation pacing threshold cut-offs to predict a successful ablation lesion. Odds ratios (OR) and 95% confident intervals (CI) of the OR are also provided

Parameter	Sensitivity	Specificity	PPV	NPV	Accuracy	OR	95% CI
ME electrogram voltage > 0.33 mV	78%	78%	91%	54%	78%	12.1	4.2–34.4
Pacing threshold < 1.5 mA	60%	59%	81%	33%	60%	2.2	0.9–5.3
Electrogram voltage > 0.33 mV and pacing threshold < 1.5 mA	52%	85%	91%	38%	61%	6.3	2.0–20.0

**Fig. 6:**
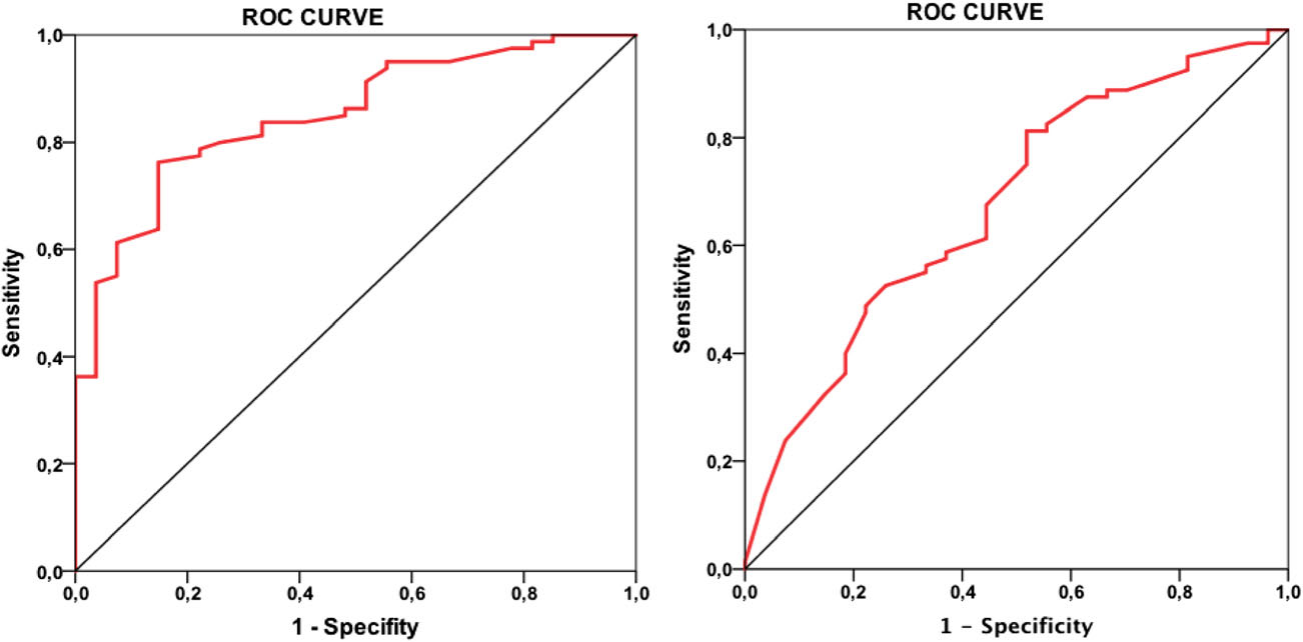
ROC curves for the mini-electrode pre-ablation electrogram voltage (left panel) and pacing threshold (right panel)

The ROC curve for the pre-ablation voltage of the ME bipole showed an AUC of 0.85 (95% CI 0.77–0.93; *P* < 0.001, Fig. 6). When the cut-off value is defined as the nearest point to the top left corner of the ROC curve, the value was 0.33 mV, and higher voltages predicted a successful lesion with a sensitivity of 78% and a specificity of 78% with a PPV of 91% and a NPV of 54% (Table 4).

For the pre-ablation pacing threshold, the ROC curve showed an AUC of 0.67 (95% CI 0.56–0.79; *P* = 0.006, Fig. 6). In the same way, for the pre-ablation threshold, the nearest point to the top left corner of the ROC curve was 1.5 mA; lower thresholds predicted a successful lesion with a sensitivity of 60% and a specificity of 59% with a PPV of 81% and a NPV of 33% (Table 4).

The combination of the two cut-off values (pre-ablation ME voltage > 0.33 mV and pre-ablation ME pacing threshold < 1.5 mA) predicted a successful lesion with a sensitivity of 52% and a specificity of 85%. The PPV in this case was 91% and the NPV was 38% (Table 4).

Overall, pacing capture during RF applications was lost in a mean of 9.9 ± 14.9 s. Time to loss of capture was not significantly different in successful in comparison to unsuccessful (9.9 ± 16.1 vs 9.7 ± 8.6 s, *P* = 0.9, Table 3).

Since power is an important determinant of lesion size, and with the use of non-irrigated catheters, delivered power is frequently different from selected power, we investigated the relationship between delivered power and electrogram voltage or pacing threshold. The mean power delivered for successful lesions was 23.9 ± 13.2 W, and for unsuccessful lesions, it was 24.7 ± 15.9 W (*P* = 0.8, Table 3). There was no correlation between delivered power and electrogram voltage (Spearman Rho, *r* = − 0.069, *P* = 0.5, Pearson, *r* = 0.044, *P* = 0.7) or pacing threshold (Spearman Rho = 0.043, *P* = 0.7, Pearson *r* = 0.05, *P* = 0.6).

We performed two sub-group analyses. One included analysis of instances of CTI block after the first pass indicative of an electrophysiologic effect, suggesting efficacy of at least the majority of the lesions. CTI block after the first ablation line occurred in 4 patients, involving 14 radiofrequency applications. Just as a reference, these 14 applications were analyzed separately and 64% and 87% of them fulfilled our voltage or threshold criteria of a successful lesion. Thus, most lesions would have been predicted to be successful according to our cut-off values for a successful lesion.

A second subanalysis was based on the rhythm at the time of ablation whether it was flutter or sinus rhythm (Table 5). This analysis showed that there were no statistically significant differences in the mini-electrode pre-ablation voltage or threshold or in the changes of these parameters after ablation in relation to the patient’s rhythm (Table 5).

**Table 5:** Electrogram voltages and stimulation thresholds in relation to the rhythm at the time of ablation

	Sinus rhythm (*n* = 30)	Atrial flutter (*n* = 77)	*P*
Pre-ablation electrogram voltage (mV), mini-electrodes	0.77 ± 0.73	0.62 ± 0.53	0.2
Pre-ablation electrogram voltage (mV) conventional bipole	0.58 ± 0.36	0.49 ± 0.22	0.1
Pre-ablation pacing threshold (mA), mini-electrodes	1.65 ± 1.89	2.5 ± 2.67	0.1
Pre-ablation pacing threshold (mA) conventional bipole	4.12 ± 2.61	6.03 ± 3. 65	0.01
Percent reduction (pre-post ablation) in electrogram voltage	66.13 ± 26.43	68.03 ± 26.15	0.7
Percent increase (pre-post ablation) in pacing threshold	85.8 ± 25.84	87.35 ± 13.49	0.7

## Discussion

Using a catheter with ME embedded in an 8-mm tip, the following observations were made during the delivery of RF current to atrial tissue in the CTI area which comprise the main findings of the present study: (1) standard RF applications produced a higher decrease in electrogram voltage and a higher increase in pacing threshold at the ME bipole than with the conventional tip to ring bipole; (2) a higher electrogram voltage and a lower pacing threshold with ME predicted a successful lesion, as defined using criteria derived from preclinical studies in atrial tissue. In contrast, neither of these parameters derived from the conventional tip to ring bipoles nor time to loss of capture during pacing at the ME had significant predictive values.

### Successful vs unsuccessful lesions

One of the limitations in the study of energy applications in clinical electrophysiology is that it is unknown if atrial lesions are transmural or not. Several studies have shown, using electrodes of conventional size in animal models, that there is a relationship between the decrease in signal amplitude and the likelihood of the lesion being transmural. Avitall et al., using multipolar catheters designed to produce linear lesions, showed that right atrial lesions were less likely to be transmural (63% transmural) when the bipolar electrogram amplitude decreased by < 50%, compared to when the amplitude decreased ≥ 50% (88% transmural) [1]. Gepstein et al. used an increase in power until the unipolar electrogram amplitude decreased by 80%, showing that all 130 right atrial lesions in 8 pigs were transmural [4]. Sanchez et al. produced 7 linear lesions (6 electrodes on each catheter) in the right atrium of 7 sheep. Lesions that were transmural resulted in a greater decrease in electrogram amplitude as recorded with both unipolar (49 ± 18% decrease in transmural vs 15 ± 20% in non-transmural lesions, *P* < 0.001) or bipolar recordings (63 ± 17% decrease in transmural vs 42 ± 19% in non-transmural lesions, *P* = 0.002) [5]. Thus, there is clear experimental evidence of the relationship between decrease in amplitude of electrical signals and atrial lesion transmurality.

Signals derived from ME have also been studied in animal models. Jumrussirikul et al. studied signals derived from a 3.7-Fr microcatheter before and after right atrial lesions, 45% of which were transmural [6]. The unipolar amplitude decreased by 36 ± 37% in transmural lesions vs 8 ± 55% in non-transmural lesions. More recently, Avitall et al. studied, in a canine model, bipolar signals of ME embedded in a 4- or 8-mm tip catheter as well as conventional bipolar signals from the regular catheter tips [7, 8]. In their model, RF lesions were transmural in 95% of the applications. After 1-min applications, conventional bipolar electrogram amplitude of the 8-mm tip electrode decreased by 43 ± 24%. In contrast, bipolar electrogram amplitude of the ME decreased by 82 ± 14% [3]. This dramatic decrease in the bipolar amplitude of the ME of more than half the amount of the conventional bipoles prompted us to consider this parameter as our gold standard for “successful” (presumably transmural) lesions, when decreases in amplitudes exceeded 54% (2 SD of the mean value to include 95% of the observations). A potential concern could be the analysis of a parameter (pre-ablation electrogram voltage) when the “gold standard” is involved somehow with the same biological variable. In order to avoid a potential bias, the “gold standard” was expressed as a percentage, i.e., corrected for the initial value. A similar methodology has been used in previous studies [5, 6].

### ME and conventional signals

The unique design of the catheter, with ME embedded in the conventional 8-mm tip, allowed for the comparison of recorded signals with different configurations (conventional vs ME) but originating from the same area. Consistent with the results of the preclinical study of Avitall et al. [3] in our clinical study, the decrease of the ME bipolar voltage in relation to RF applications exceeded that of the conventional bipole (Fig. 1).

Furthermore, the RF-related increase in the ME bipolar pacing threshold exceeded that of the conventional bipole (Fig. 1). Both findings are probably related to a higher far-field content and virtual electrode size of a bipole generated by two poles 11.5 mm away (distance between the tip of the ablation electrode and the proximal end of the ring) in comparison to that of two 0.8-mm electrodes with 1.2-mm interelectrode distance that are located inside the electrode which creates the lesion. Intuitively, parameters generated at the core at which the lesion is formed, at that undergo a more intense change with the RF application would be more likely to reflect the lesion that is being produced clinically.

The pre-ablation amplitude of the ME bipolar signal was significantly larger than that of the conventional bipole, but the mean amplitude of each was well below 1 mV. This is in contrast to the findings in the preclinical study of Avitall et al. [3], but consistent to the clinical study of Lloyd et al. [9]. In the former, the mean amplitude of the conventional bipolar signals approximated 4 mV whereas that of the ME was 2.4 mV. The reason for this difference is not totally clear. Data from the preclinical study came from normal dogs whereas our clinical data and that from Loyd et al. came from patients with atrial flutter. It is conceivable that the diseased atria of our patients generated signals of lower voltage.

This could imply some degree of inaccuracy in the cut-off used to consider a lesion as successful. But on the other hand, since the parameter of electrogram voltage contains biological information about tissue characteristics, it is likely that proportional cut-offs may remain. It is also conceivable that in moving the catheter positions for ablation, we selected sites with a relatively large local ME signal that could have less far-field signals because they were surrounded by diseased tissue.

However, our data for pre-ablation pacing thresholds were consistent with that of the preclinical study [10]. Pacing thresholds of the ME, both in our study and in the preclinical study, were lower than those of the conventional bipole. The higher current density generated by the ME needing less current to excite the myocardium may explain this finding. The absolute value of the mean pacing thresholds in our study was approximately double than those obtained in the preclinical study. This supports our previous suggestion that atria of patients with atrial flutter is less healthy and thus less excitable that the healthy atria of the animals of the preclinical study.

### Pre-ablation parameters and transmural lesions

The data relating decrease in amplitude of electrical signals and atrial lesion transmurality is clear [1, 3, 4, 6] and frequently used in clinical grounds to assess if a lesion is successful. Lack of pacing capture over ablated tissue has also been demonstrated to be related to successful lesions [11]. However, those parameters can only be obtained after lesion delivery and it would be desirable to predict lesion adequacy before its deployment in clinical electrophysiology.

We demonstrate for the first time that pre-ablation bipolar amplitude and pacing threshold, as obtained with the ME, were significantly different for successful vs unsuccessful lesions, and in fact, an amplitude > 0.32 mV and a pacing threshold < 1.5 mA predicted a successful lesion. Consistent with our findings, in a preclinical study with a different type of ME [6], pre-ablation unipolar pacing threshold was also shown to differ in transmural vs non-transmural lesions. Notably, mean values of transmural vs non-transmural lesions were quite similar to the ones we obtained in our population (1.7 vs 3.1 mA in the preclinical study and 1.9 vs 4.7 in our study). The reason for these findings could be mediated by catheter contact force, which has been shown to be a major determinant of lesion size, and related to pacing threshold [8, 10, 11]. Using intracardiac echo [12] to assess contact, ME bipolar amplitude was shown to correlate with catheter endocardial contact [13]. Thus, it is likely that better contact force produced higher electrogram amplitudes and lower pacing thresholds often resulting in a successful lesion, although these relationships could not be obtained because the catheter lacks contact force measurements. The interest of developing parameters that could be related to contact force could be a matter of debate. However, it should be acknowledged that contact force is not without limitations and it entails a higher cost; contact force does not take into consideration tissue characteristics or catheter stability. A recent study found that local impedance had a superior fit to lesion dimension than models that incorporated force time integral [14]. Thus, it is worth exploring if other parameters could also be of value in determining a successful lesion. Although we cannot propose using these measurements as an alternative to contact force without the support of additional basic and clinical studies, the finding that parameters derived from mini-electrodes could offer some value when conventional electrodes do not, is of potential importance. For example, if parameters are not optimal at an anatomically appropriate site, small movements of the catheter can improve the parameters.

It should be mentioned that when similar parameters were obtained from conventional bipoles, no significant differences were observed between successful and unsuccessful lesions, again attesting to the limitations derived from the far-field signal content and size of virtual electrode of these configurations.

In contrast with the above findings and despite our initial hypothesis, we did not observe any relationship between the time to loss of capture while pacing during ablation and the appearance of successful lesions. It is conceivable that the relationship between pacing threshold, pacing output, virtual electrode size, and lesion formation is more complex than we had anticipated.

Ultimately, a successful lesion results from a complex interplay of tissue volume, catheter stability, contact, tissue characteristics, and power delivery. Factors such as voltage/voltage drop, impedance/impedance drop, pacing threshold/change in thresholds are all surrogates for these.

### Limitations

Since this is a clinical study, obviously there is no histologic evidence of the lesions created, and our “gold standard” was derived from preclinical information. Although ideally, it would have been desirable to obtain an additional estimation of lesion size by a different technology, no accurate technology existed to be used in clinical electrophysiology at the time of the study. Intracardiac echocardiography has been used in some studies, but it was found unable to precisely delineate lesion dimensions [15]. Generator impedance drop has been shown to correlate poorly with lesion depth and even worse with lesion diameter [14]. Local impedance appears to be a reliable indicator of lesion formation [14] but was not available at the time of the study.

We tried to avoid lesions created on tissue that already had previous ablations because the relationship between voltage and pacing threshold with lesion formation could presumably be different. For that reason, we only considered the applications of the initial ablation line as the catheter was pulled back from a position close to the tricuspid annulus to a position close to the inferior vena cava. However, we cannot completely exclude that some lesions could have involved partially damaged tissue.

Catheter stability is crucial for measurements after ablation to explore the same tissue tested before ablation. Although this was assessed by experienced operators using fluoroscopy and lesions were excluded from analysis whenever there was a suspicion of catheter instability, the limitations of this methodology should be acknowledged.

Since power is an important determinant of lesion size and with non-irrigated catheters delivered power is frequently different from selected power, this could be an additional uncontrolled factor influencing the relationship between electrophysiological parameters and lesion size. However, the absence of correlation between delivered power and electrogram voltage or pacing threshold and the absence of significant differences of delivered power in presumably successful vs unsuccessful lesions makes it unlikely that power had a relevant influence in the observed results. This is not to say that power was always sufficient during lesion delivery. In accordance with a previous study [16], we had to change to another catheter (irrigated) in a significant number of patients, due to insufficient power output. This could have been overcome with the use of an irrigated tip catheter, but unfortunately, an irrigated tip catheter with mini-electrodes was not available at the time of the study.

We paced from the mini-electrode bipole with the largest electrogram amplitude. It is not clear if the mini-electrode bipole with the largest electrogram amplitude has the lowest pacing threshold. We studied this in a small series of four additional patients with atrial flutter and found a negative but weak correlation between electrogram voltage and pacing threshold (−Pearson *r*: − 0.3, *p*: 0.025, −Spearman rho: − 2.5, *p*: 0.06). The bipole with the highest voltage had the lowest threshold in only 41% of sites. Thus, it is conceivable that pacing threshold could have predicted a successful lesion even better if the bipole with the lowest threshold had been used. However, this methodology will increase the duration of the procedure.

This study was performed during clinical attempts at CTI ablation. The extent to which the observed results apply to other atrial regions is unknown.

## Conclusions

The influence of RF applications on electrophysiologic parameters such as bipolar electrogram voltage and pacing threshold was found to be more intense when those parameters are derived from ME embedded in an 8-mm tip as opposed to conventional bipoles.

In contrast to the observations from conventional bipoles, pre-ablation ME electrogram voltage and pacing threshold independently predicted a successful lesion, as defined using criteria derived from preclinical studies in atrial tissue.

These parameters could be used prior to lesion delivery and could likely improve lesion quality.
